# Vertebral hyperostosis, ankylosed vertebral fracture and atlantoaxial rotatory subluxation in an elderly patient with a history of infantile idiopathic scoliosis; a case report

**DOI:** 10.1186/1752-1947-1-25

**Published:** 2007-06-06

**Authors:** Ali Al Kaissi, Elisabeth Zwettler, Katharina M Roetzer, Joerg Haller, Franz Varga, Klaus Klaushofer, Franz Grill

**Affiliations:** 1 Ludwig Boltzmann Institute of Osteology at the Hanusch Hospital of WGKK and AUVA Trauma Centre Meidling, 4th Medical Department, Hanusch Hospital, Vienna, Austria; 2 Orthopaedic Hospital of Speising, Vienna, Austria

## Abstract

This is a case report of a 48-year-old-woman with scoliosis since early childhood. Recent radiographic spinal assessment revealed the presence of distinctive spinal abnormalities. To the best of our knowledge this is the first clinical report describing a constellation of unusual changes in an elderly woman with a history of infantile idiopathic scoliosis.

## Case presentation

Forestier and Rotes-Querol first described the disease Diffuse Idiopathic Skeletal Hyperostosis (DISH) in 1950 [1]. These authors provided a precise description, separating the disease from discoarthrosis and ankylosing spondylitis. Resnick and Niwayama [2] described the diffuse nature of the disease and proposed widely used diagnostic criteria. The disease is usually seen in male patients over 45 years of age and characterised by new bone formation at the entheses. Diagnostic criteria of DISH include flowing ossification along at least 4 contiguous vertebrae, preservation of disk spaces, absence of vacuum phenomena or vertebral body marginal sclerosis, and absence of apophyseal joint ankylosis or sacroiliac joint erosions or fusion. The thoracic spine is most commonly involved, but radiographic findings in both the spine and extraspinal structures suggest a generalised disorder of ossification rather than a localised spinal disease. While Diffuse Idiopathic Skeletal Hyperostosis (DISH) is mostly asymptomatic, it can predispose the patient to catastrophic complications. The common potential complications of DISH in the cervical and thoracic spine include fractures, dysphagia, cervical and/or thoracic myelopathy, paraplegia, and dens spinal cord injury resulting from even minor trauma [1-6].

On the other hand the classical risk factors for DISH are known to co-exist in the following conditions; diabetes mellitus type 2, obesity, and hyperuricaemia [6]. Previous reports describing DISH patients with spinal fractures are rare [3-5]. Scoliosis is seemingly not listed among the major risk factors for the development of DISH. The purpose of this case report is to characterise and consider whether idiopathic infantile scoliosis represents an additional risk factor for the development of spinal hyperostosis in elderly people

**Figure 1:**
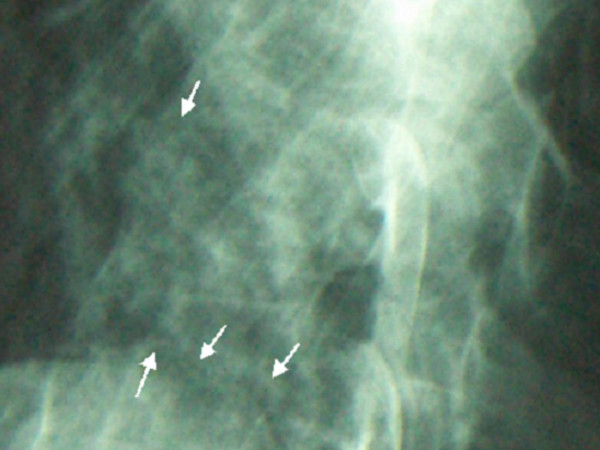
Ankylosed spine fractures in lateral radiogram of the thoracic vertebrae, arrows showed sawtooth aspect fractures.

**Figure 2:**
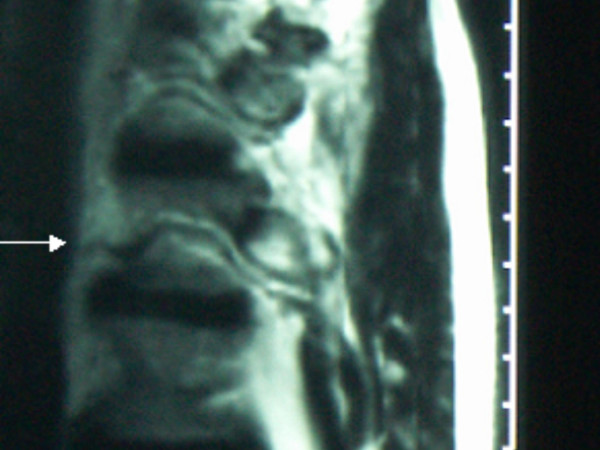
Sagittal MRI shows progressive simultaneous ossification of the anterior longitudinal and the posterior longitudinal spinal ligaments respectively and the apparent ankylosed spine fracture (arrow).

The patient has a history of idiopathic infantile scoliosis. Her scoliosis was treated at the age of 9 years by bracing technique only. Apparently, the nature of her scoliosis was progressive; her Cobb's angle was 64°. The patient was recently identified as having spinal osteoporosis. The periodic clinical and radiographic assessment revealed the diagnosis of diffuse idiopathic skeletal hyperostosis (fig 1, 2)

She was married and her gestational history revealed five pregnancies (two spontaneous abortions in the first trimester and another two ectopic pregnancies, the reason behind these events were not identified). She had one normal male offspring.

**Figure 3:**
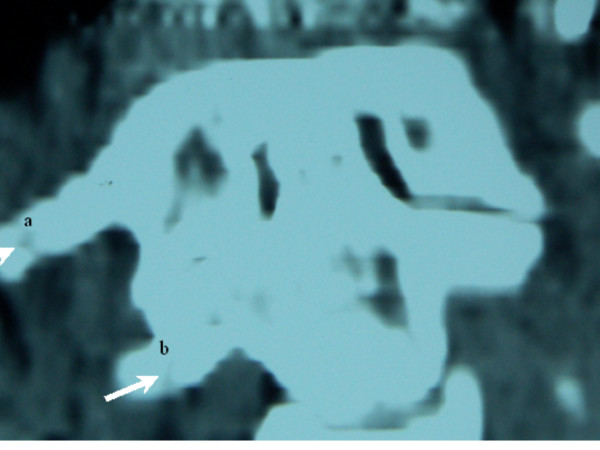
Coronal computerised tomographic reconstructions demonstrating asymmetric lateral atlantodental intervals because of rotation at C1-C2. Note C2 in the coronal position with the anterior arch of C1 markedly overlying the right facet resulting in AARS and the existence of two fractures; a) healed fracture of the right anterior arch of atlas and b) another healed fracture of the lateral right process of C2.

Clinical examination revealed normal phenotype, thin woman with significant thoracic kyphoscoliosis. Passive rotation of the neck was associated with moderate pain. There was mild torticollis (fig 3). Tenderness along the cervical area was noted. Similarly point tenderness over the upper thoracic spine was notable (fig 1). A neurologic examination included testing muscle strength, sensation and reflexes of the lower extremities. Bowel and bladder sphincter control were all within normal limits. No complaint of pain in her pelvis. Examining the rest of her joints revealed nothing of significance. Her height, weight and head circumference were normal. Abdominal and renal ultrasound was normal.

Recently, she was identified, as being affected with spinal osteoporosis through the national screening programme. Central (DXA) showed that lumbar spine, T -Score -3.1 SD (osteoporosis), whereas the femoral neck T-Score -1.7 SD (osteopenia). Blood sugar and uric acid levels were normal. Biochemical tests showed mild elevations of serum calcium 2.74 (2.20–265 mmol/l), ionised calcium 1.63 (1.18–1.30 mmol/l), β-crosslaps 1.170 (normal for postmenopausal women is 0.400–1.008 ng/ml).

Diffuse idiopathic skeletal hyperostosis (DISH) is an ossifying, non-inflammatory, non-erosive enthesopathy favouring the dorsal spine but sparing the sacroiliac joints. DISH affects 3–6% of the population over 40 years of age and 11% aged over 70 years [1-4]. A varying proportion of patients with DISH have ossification of the posterior longitudinal ligaments.

DISH leads to acquired narrowing of the spinal canal due to the presence of osteophytes, which sometimes create bony hooks within the spinal canal [1-6]. The cause and pathogenesis of DISH are still unknown. Kiss et al., [5] studied the risk factors and the radiographic features of 131(69 males and 62 females) affected with DISH. Obesity, diabetes mellitus, smoking, and hypertension were shown to be the most likely predisposing factors. Some authors have noted the occasional familial incidence of DISH, leading to a suspicion of genetic predisposition [6]

De Peretti et al [4], described 48 fractures in 48 patients over a period of 17 years. Twenty patients (mean age 62 years) had ankylosing spondylitis and 28 patients (mean age 81 years) had DISH syndrome. They concluded that spinal fractures in patients with DISH syndrome generally occur spontaneously or after low-energy trauma. None of the reported patients showed a history of scoliosis. They identified 4 types of spine fractures in their series. Our patient manifested type II fracture of the de Peretti et al, classification.

Previous studies have indicated a significant correlation of osteoporosis with idiopathic scoliosis in adults. Cheng and Guo [7] supported the hypothesis that adolescents with idiopathic scoliosis are at increased risk of osteoporosis compared to the general paediatric population. None of these reports signified the correlation between idiopathic infantile scoliosis and the development of DISH later in life.

Our patient illustrated lateral displacement of the dens by more than 4 mm, the latter was suggestive of atlanto-axial rotatory subluxation (AARS) [8]. The computerised tomogram of the atlanto-axial region demonstrated asymmetrical odontoid-lateral mass distance and confirmed the existence of two healed fractures. It is suggested that the persistence of abnormal dynamics, secondary to the delay in treating AARS, can lead to the development of pathological stickiness between the atlas and the axis, probably because of contractures of peri-articular soft tissue [8,9]. Evidence supports, that conservative treatment in the elderly population can be managed non-operatively, because few demands are made on the neck. In young adults the story is different. The use of early cranial traction followed by external immobilisation for six weeks is a usual procedure to achieve good long-term stability. If recurrent or irreducible subluxation developed, open reduction and posterior atlantoaxial fusion may be required [10]

Osteoporotic vertebral compression fractures are a major cause of morbidity and health care cost among elderly patients. In the past, the primary therapy for these fractures has been conservative. Percutaneous vertebroplasty is now a therapeutic option for individuals where medical management has not been successful or for those at risk of developing complications due to long-term immobilization. On the other hand, injectable biomaterials may decrease the incidence of new vertebral fracture e.g. calcium phosphate has been introduced to relieve pain and at the same time is capable of integrating into the bony matrix [11]

## Conclusion

In the light of our findings, we believe that the degree of osteoporosis of the adjacent vertebral bodies, the development of ankylosed spinal hyperostosis, fractures and AARS are a constellation of abnormalities developed in connection with the early onset of idiopathic infantile scoliosis. DISH patients are usually reported to have a history of diabetes mellitus, obesity, and hyperuricaemia. We wish to stress, that scoliosis might be a possible confounder in the relationship between spinal osteoporosis and the development of spinal hyperostosis in the elderly. Further studies are needed to elucidate this sort of correlation.

## Abbreviations

(DISH) diffuse idiopathic skeletal hyperostosis; (AARS) Atlantoaxial rotatory subluxation; (AAS) atlanto axial subluxation.

## Competing interests

The author(s) declare that they have no competing interests.

## Authors' contributions

All authors read and approved the final manuscript and all participate in this work.
